# Triple RISC-Assisted Exciton-Harvesting System for Efficient White Organic Light-Emitting Diodes

**DOI:** 10.3390/mi17070856

**Published:** 2026-07-17

**Authors:** Yali Li, Shuming Chen, Jintao Wang

**Affiliations:** 1 School of Information Engineering, Yantai Institute of Technology, Yantai 264000, China; 2 College of Physics, Changchun University of Science and Technology, Changchun 130000, China

**Keywords:** WOLED, thermally activated delayed fluorescence, exciplex, charge balance

## Abstract

Developing white organic light-emitting diodes (WOLEDs) with high exciton utilization, balanced charge transport, and stable complementary emission remains a challenge for solid-state lighting and display applications. Herein, a triplet reverse intersystem crossing (RISC)-assisted strategy is proposed to enhance triplet exciton harvesting to construct efficient hybrid WOLEDs. The increased RISC channels promote the up-conversion of triplet excitons into radiative singlet excitons, thereby improving the overall exciton utilization efficiency. By further introducing an ultrathin PO-01 layer as an orange orange-emitting component, a hybrid WOLED with a current efficiency of 49.1 cd/A and 34.8 lm/W is realized. Moreover, suppressed efficiency roll-offs and stable spectra are achieved due to balanced charge transport. This work provides a practical route toward high-performance WOLEDs.

## 1. Introduction

Organic light-emitting diodes (OLEDs) have attracted extensive attention owing to their advantages of self-emission, thin-film processability, mechanical flexibility, and large-area fabrication [1,2]. Among them, white OLEDs (WOLEDs) are particularly promising for solid-state lighting and full-color display technologies [3,4,5]. In conventional fluorescent WOLEDs, only singlet excitons can be directly harvested for light emission, whereas electrically generated triplet excitons are lost via nonradiative pathways [6]. Phosphorescent emitters can utilize both singlet and triplet excitons through strong spin–orbit coupling, but their reliance on noble-metal complexes and stability issues motivate the development of metal-free alternatives [7,8,9]. Hybrid WOLEDs constructed with phosphorescence and thermally activated delayed fluorescence (TADF) provide a practical and viable pathway [10,11]. TADF materials with reverse intersystem crossing (RISC) convert triplet excitons into singlet excitons due to the small singlet–triplet energy gap, making them a powerful technology for improving exciton utilization in WOLEDs [12,13].

Exciplex systems formed between electron-donating and electron-accepting molecules are especially attractive because their charge-transfer excited states can exhibit small singlet–triplet energy splitting and efficient RISC [14,15,16]. In addition, exciplex hosts provide balanced bipolar charge transport, which broaden the exciton recombination zone and reduce exciton quenching. These features are highly desirable for WOLEDs, where efficient exciton harvesting and controllable energy transfer to complementary emitters are required [17,18,19]. Recently, Zhang et al. reported highly efficient hybrid WOLEDs based on blue exciplex emission, achieving maximum efficiencies of 55.3 cd/A (46.8 lm/W) [20]. Lan et al. reported high-performance cool WOLEDs with a high color rendering index, achieving maximum efficiencies of 34.4 cd/A (36.1 lm/W) [21]. Nevertheless, the utilization efficiency of triplet excitons via a single RISC channel remains constrained. Consequently, enhancing the overall exciton utilization is essential for further performance improvements of hybrid WOLEDs. Specifically, the simultaneous realization of high efficiency, stable white emission, and simple device architecture remains challenging.

In this work, a triplet RISC-assisted emitting system is constructed by integrating 26DCzPPY, DMAC-DPS, and B4PyPPM in an emitting layer. The coexistence of these RISC channels enables efficient triplet-to-singlet up-conversion and offers multiple pathways for exciton harvesting. Moreover, the bipolar transport property of exciplex hosts reduces triplet exciton accumulation in the EMLs, leading to suppressed efficiency roll-off. On this basis, an ultrathin PO-01 layer is introduced in combination with RISC-assisted blue emitters to achieve hybrid WOLEDs. The resulting WOLEDs show a high current efficiency of 49.1 cd/A and a power efficiency of 34.8 lm/W, with a low turn-on voltage of 2.4 V. The Commission Internationale de l’Eclairage (CIE) coordinates show a small change (0.001, 0.003) with a voltage range of 5 V to 8 V, thus exhibiting stable emission spectra. These results reveal a rational basis for the fabrication of efficient WOLEDs.

## 2. Experimental Section

All organic materials were purchased commercially. 26 DCzPPY was employed as a hole-transporting donor component, B4 PyPPM was used as an electron-transporting acceptor component, and DMAC-DPS served as a TADF-active donor component. PO-01 was selected as the orange-emitting ultrathin layer to construct complementary white emitters. The above materials were purchased from Xi’an Yuri Solar Co., Ltd., Xi’an, China).

OLEDs were fabricated on patterned indium tin oxide (ITO)-coated glass substrates (purchased from Liaoning Youxuan New Energy Technology Co., Ltd., Yingkou, China). The substrates were sequentially cleaned by ultrasonication in detergent solution, deionized water, acetone, and isopropanol, followed by drying and ultraviolet–ozone treatment. The organic functional layers and metal cathodes were deposited by thermal evaporation under high vacuum. The deposition rates of the organic layers and metal electrodes were monitored by quartz crystal sensors. The electroluminescence (EL) spectra, current density–voltage–luminance characteristics, current efficiency, power efficiency, external quantum efficiency, and CIE coordinates were measured by a computer-controlled Keithley 2400 source (Tektronix Inc., Beaverton, OR, USA) integrated with a PR655 spectrometer (Photo Reserch Inc., Simi Valley, CA, USA). The PL spectra were acquired by an RF-5301 PC fluorescence spectrophotometer (Shimadzu, Kyoto, Japan). The transient PL decay curves were recorded by an IHR320 spectrometer (HORIBA, Kyoto, Japan).

## 3. Results and Discussion

**Figure 1:**
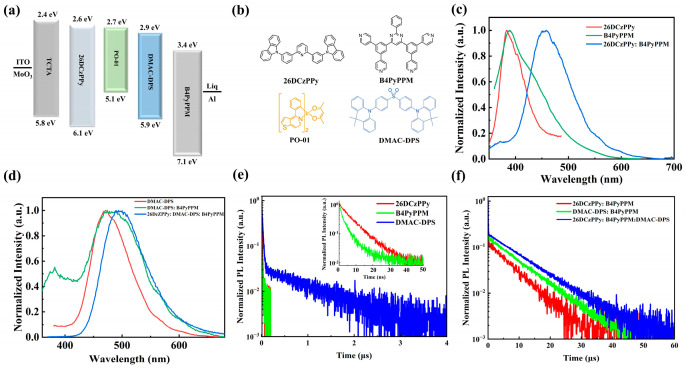
(**a**) Energy levels of used materials. (**b**) Chemical structures of materials used. (**c**) PL spectra of 26DCzPPY, B4PyPPM, and mixed films. (**d**) PL spectra of DMAC-DPS, DMAC-DPS: B4PyPPM, and 26DCzPPY: B4PyPPM: DMAC-DPS mixed films. (**e**) Transient PL decay curves of 26DCzPPY, B4PyPPM, and mixed films. (**f**) Transient PL decay curves of DMAC-DPS, DMAC-DPS: B4PyPPM, and 26DCzPPY: B4PyPPM: DMAC-DPS mixed films.

The molecular chemical structures and energy levels of the used molecules are illustrated in Figure 1a,b. The PL spectra of the individual and mixed films are shown in Figure 1c. The neat films of 26DCzPPY and B4PyPPM shows emission peaks at 380 nm and 389 nm, and the mixed films show redshifts compared to the neat films. The 26DCzPPY:B4PyPPM blend film with a 1:1 ratio exhibits a blue emission band with a peak at 461 nm, which can be assigned to the exciplex formed between the donor of 26DCzPPY and the acceptor of B4PyPPM. As shown in Figure 1d, DMAC-DPS shows emission peaks at 471 nm. Specifically, the DMAC-DPS: B4PyPPM blend film shows an emission peak at 482 nm, indicating the formation of a charge-transfer excited state between DMAC-DPS and B4 PyPPM. Whereas the peak of 380 nm observed in the DMAC-DPS: B4PyPPM film, originating from B4PyPPM emission, is owed to the low concentration of its donor DMAC-DPS. As for the 26DCzPPY: B4PyPPM: DMAC-DPS ternary film, only one peak of 493 nm is shown without additional emission peaks, inferring sufficient formation of the DMAC-DPS: B4PyPPM exciplex. It is noted that the partial acceptor of B4PyPPM molecules can form exciplexes with 26DCzPPY and then transfer energy to DMAC-DPS: B4PyPPM exciplexes, leading to efficient energy transfer from high-energy exciplexes to low-energy exciplexes.

As shown in Figure 1d, time transient PL decay measurements are used to further confirm the TADF behavior and energy transfer process of mixed films. Obviously, the three mixed films of 26DCzPPY: B4PyPPM, DMAC-DPS: B4PyPPM, and 26DCzPPY: B4PyPPM: DMAC-DPS exhibit longer lifetimes compared to pure individual films. Meanwhile, the 26DCzPPY: B4PyPPM mixed film shows a short lifetime (τ_PF_) of 38.4 ns and a long lifetime (τ_DF_) of 6.86 μs, which represents the prompt fluorescence and delayed fluorescence, respectively. The DMAC-DPS: B4PyPPM film shows a prompt component of 39.4 ns and a delayed component of 8.58 μs, demonstrating longer lifetime compared with those of DMAC-DPS (19.2 ns for τ_PF_ and 1.1 μs for τ_DF_) due to the introduction of an additional RISC process of exciplexes. The ternary film of 26DCzPPY: B4PyPPM: DMAC-DPS shows the longest lifetime of the three films, with a prompt component of 46.5 ns and a delayed component of 9.76 μs, resulting from triple RISC channels in the film. The exciton dynamic process is further discussed in Table 1. As RISC channels increase, PLQY of the three films increase obviously from 23% to 57%, which infers improved exciton utilization in the films. K_RISC_ represents the rate of the RISC process and is further calculated [22,23]. The 26DCzPPY: B4PyPPM: DMAC-DPS film shows an improved K_RISC_ of 5.08 × 10^6^ s^−1^, which is higher than that of DMAC-DPS: B4PyPPM films at 4.84 × 10^6^ s^−1^, inferring improved triplet exciton utilization in the ternary film.

**Figure 2:**
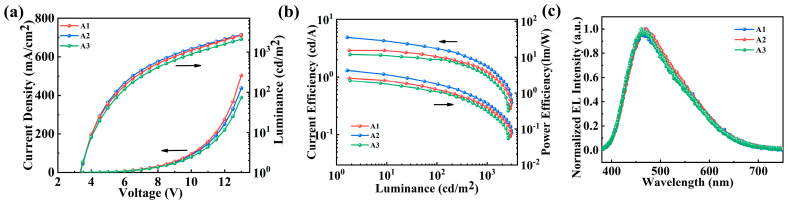
(**a**) Current density–voltage–luminance curves of devices A1–A3. (**b**) Current efficiency–luminance–power efficiency curves of devices A1–A3. (**c**) Normalized EL spectra of devices A1–A3 at 6 V.

To elucidate the EL performance characteristics of the 26DCzPPY: B4PyPPM exciplex system, we constructed a series of devices with the following structure: ITO/MoO_3_ (2 nm)/TCTA (45 nm)/26DCzPPY (5 nm)/26DCzPPY: B4PyPPM (25 nm)/B4PyPPM (35 nm)/Liq (1 nm)/Al. The ratios of 26DCzPPY to B4PyPPM are adjusted to 1:1, 1:2, and 1:4 for devices A1, A2, and A3, respectively. The current density–voltage–luminance characteristics are depicted in Figure 2a and indicate that the turn-on voltages for devices A1-A3 are approximately 3.3 V. These low turn-on voltages suggest a minimal energy barrier within the organic layers, facilitating efficient charge carrier injection and transport at a lower electric field. It is noted that there is a decline in current density as the B4PyPPM doping concentration increases from devices A1 to A3. This trend is attributed to B4PyPPM’s enhanced electron transport capabilities relative to the hole transport properties of 26DCzPPY. As shown in Figure 2b, the current efficiencies and power efficiencies of the four devices shows a trend of first increasing and then decreasing. Device A2 achieves the highest current efficiency and power efficiency among the four devices, with values of 4.87 cd/A and 4.27 lm/W, respectively. It can be explained that as B4PyPPM concentration increases, the electron transport mobility is enhanced and realizes balanced charge carrier transport in device A2. Figure 2c reveals that all devices exhibit a single electroluminescence (EL) peak at approximately 460 nm at 6 V, which is consistent with the PL peak.

**Figure 3:**
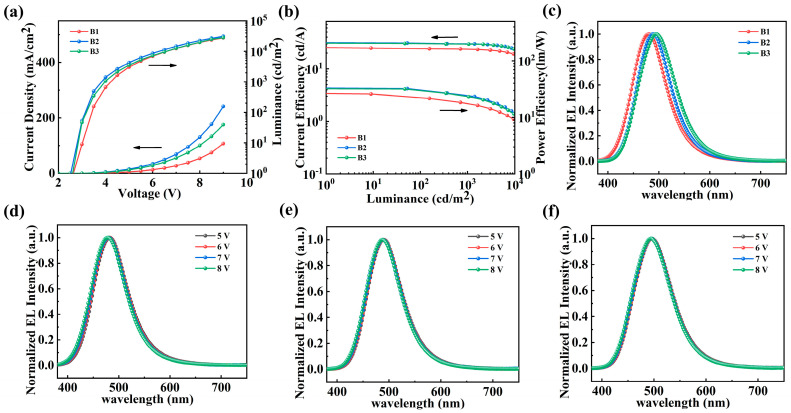
(**a**) Current density–voltage–luminance curves of devices B1–B3. (**b**) Current efficiency–luminance–power efficiency curves of devices B1–B3. (**c**) Normalized EL spectra of devices B1–B3 at 6 V. (**d**–**f**) Normalized EL spectra of devices B1–B3 at different voltages.

To further explore the impact of DMAC-DPS concentration on the performance of blue fluorescent devices, four devices are fabricated with the structure of ITO/MoO_3_ (2 nm)/TCTA (45 nm)/26 DCzPPY (5 nm)/26DCzPPY: B4PyPPM: DMAC-DPS (1: 2, x%) (25 nm)/B4 PyPPM (35 nm)/Liq (1 nm)/Al, x is set as 5, 15, and 25, corresponding to devices B1, B2, and B3. The current density–voltage and luminance–voltage profiles are presented in Figure 3a. The current density shows an increasing trend from devices B1 to B2, and then decreases in device B3, suggesting that DMAC-DPS has an effect on carrier transport. Meanwhile, device B2 with 15% DMAC-DPS concentration achieves the highest luminance of 30,967.5 cd/m^2^ with a turn-on voltage of 2.5 V.

The current efficiency–luminance–power efficiency curves of devices B1–B3 are shown in Figure 3b. As DMAC-DPS concentration increases, the efficiencies improve significantly first and then further reduce. Device B2 exhibits a maximum current efficiency and power efficiency of 30.9 cd/A and 32.3 lm/W, and the current efficiency still maintains 29.9 cd/A at 1000 cd/m^2^ and 27.6 cd/A at 5000 cd/m^2^, corresponding to low roll-offs of 3.2% and 10.6%. The improved efficiencies when compared to the device A series can be attributed to the enhanced RISC process. As DMAC-DPS concentration increases, more energy from triplet excitons in the 26DCzPPY: B4PyPPM exciplex can transfer to triplet excitons of DMAC-DPS through Dexter energy transfer, and then further up-convert to singlet excitons via RISC in DMAC-DPS. Finally, these singlet excitons can transfer energy to the DMAC-DPS: B4PyPPM exciplex via Förster energy transfer (FRET) and radiative emission. Therefore, by harnessing the TADF properties of DMAC-DPS, more triplet excitons that cannot emit light are converted into singlet excitons, contributing to the enhanced device performance observed in our experiments. The efficiencies of device B3 shows a declining trend owing to further increases in DMAC-DPS, which may be attributed to imbalanced charge transport.

The EL spectra of four devices are shown in Figure 3c. It can be seen that as the DMAC-DPS concentration increases, the emission peaks show noticeable redshifts from 479 nm to 494 nm at 7 V, demonstrating the emission contribution shifts from being primarily from DMAC-DPS to DMAC-DPS: B4PyPPM. Meanwhile, device B2 shows an emission peak at 487 nm, suggesting this sky-blue light emission is suitable for WOLEDs. As shown in Figure 3d–f, three devices exhibit stable spectra with increasing voltage, inferring no obvious color drift with different voltage.

**Figure 4:**
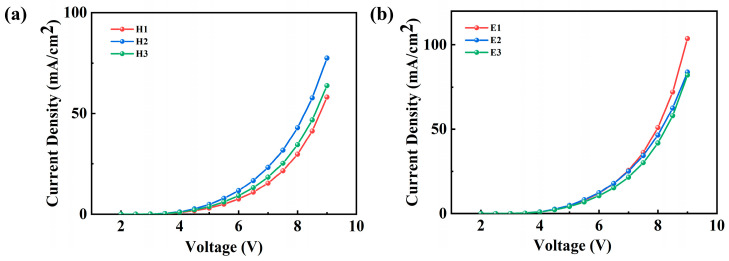
(**a**) Current density–voltage curves of devices H1–H3. (**b**) Current density–voltage curves of devices E1–E3.

The single-carrier devices are fabricated to demonstrate the impact of DMAC-DPS on charge transfer property. The device structures are ITO/MoO_3_ (2 nm)/TCTA (45 nm)/26 DCzPPy (5 nm)/26 DCzPPY: B4 PyPPM: DMAC-DPS (1: 2, x%) (25 nm)/TCTA (35 nm)/Al for hole-only devices and ITO/B4 PyPPM (45 nm)/26 DCzPPy (5 nm)/26DCzPPY: B4PyPPM: DMAC-DPS (1: 2, x%) (25 nm)/B4PyPPM (35 nm)/Liq (1 nm)/Al for electron-only devices, x is set as 5, 15, and 25, corresponding to devices H1, H2, and H3, as well as E1, E2, and E3. As depicted in Figure 4a, the hole current shows an increase as DMAC-DPS increases from 5% to 15%, and further decreases while increasing DMAC-DPS concentration. It is noted that as the concentration of DMAC-DPS increases, holes may inject into the emission layer easily due to the shallow HOMO of DMAC-DPS compared to that of 26 DCzPPy, leading to improved current density. Conversely, the hole-trapping effect becomes more pronounced with further increases in DMAC-DPS, resulting in a decreased current density [20]. The electron current exhibits a decreasing trend as DMAC-DPS increases, which can be attributed to the lower electron transport property of DMAC-DPS.

**Figure 5:**
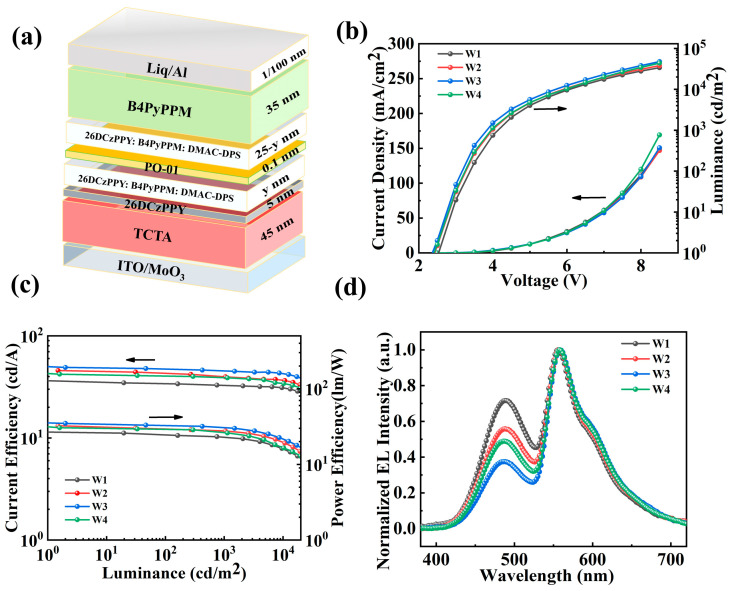
(**a**) Device structure of WOLEDs. (**b**) Current density–voltage–luminance curves of devices W1–W4. (**c**) Current efficiency–luminance–power efficiency curves of devices W1–W4. (**d**) Normalized EL spectra of devices W1–W4 at 7 V.

Based on the excellent performance of blue devices, WOLEDs are constructed by introducing a PO-01 ultrathin layer into an emitting layer, with the structure of ITO/MoO_3_ (2 nm)/TCTA (45 nm)/26DCzPPY (5 nm)/26DCzPPY: B4PyPPM: DMAC-DPS (1:2, 15%, y nm)/PO-01 (0.15 nm)/26DCzPPY: B4PyPPM: DMAC-DPS (1:2, 15%, 25-y nm)/B4PyPPM (35 nm)/Liq (1 nm)/Al—y is set as 5, 8, 12, and 18, corresponding to devices W1, W2, W3, and W4. The device structure is shown in Figure 5a; the blue emission components originate from the multiple RISC-active host systems, while the orange component is supplied by PO-01. The combination of complementary emissions results in broad white electroluminescence. As can be seen in Figure 5b, four devices show approximate current densities, indicating the influence of the ultrathin layer can be neglected. The turn-on voltages of four devices are 2.5 V, 2.5 V, 2.4 V, and 2.4 V, inferring a small injection energy barrier in the devices.

Figure 5c shows current efficiency–luminance–power efficiency curves of four devices. As can be seen, efficiencies increase from devices W1 to W3, and further decreases in W4. It is noted that as the ultrathin layer approaches the middle of the emission layer, more charge carriers recombine on PO-01 and emit light, inferring that the main exciton recombination zone is near the middle of the emission layer. Among the four devices, device W3 achieves a maximum current efficiency of 49.1 cd/A, which is 38.4% higher than that of device W1 (36.4 cd/A). Meanwhile, the current efficiency still maintains 45.5 cd/A at 1000 cd/m^2^, corresponding to low roll-offs of 7.3%, which can be attributed to an expanded exciton recombination zone in the devices. Owing to the low driving voltage, device W3 also achieves a high power efficiency of 34.8 lm/W. The efficiency enhancement should be discussed in relation to the multiple RISC-assisted exciton-harvesting mechanisms.

**Figure 6:**
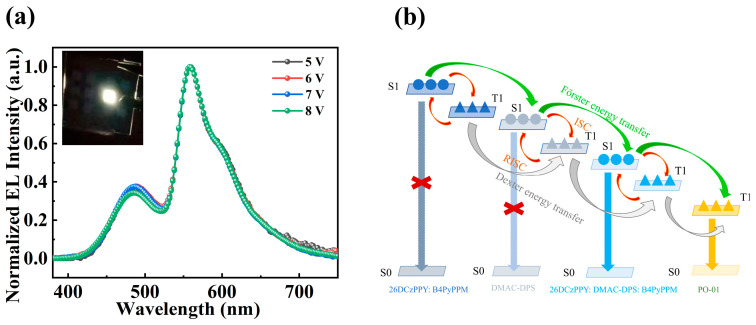
(**a**) Normalized EL spectra of device W3 at different voltages. Insert shows a photograph of the device. (**b**) Energy transfer diagram of WOLEDs.

As can be seen in Figure 5d, the EL spectra of four WOLEDs show that the white emission can be regulated by adjusting the position of the PO-01 ultrathin layer. An off-center PO-01 layer may lead to sufficient blue emission, producing a colder white color with enhanced exciplex components in device W1. Device W3 exhibits the lowest blue emission peak at 486 nm, owing to the efficient trapping effect of PO-01. The spectral stability of the optimized W3 is analyzed with different driving voltages, as shown in Figure 6a. Because the recombination zone is relatively broad and centered in the emitting layer, the device is expected to show reduced sensitivity to voltage variation. As the voltage increases from 5 V to 8 V, the CIE coordinates change from (0.397, 0.459) to (0.396, 0.456), suggesting negligible variation of (0.001, 0.003) from 1122 cd/m^2^ to 7567 cd/m^2^. The detailed summary of WOLEDs performance is shown in Table 2. It is notable that our device shows superior performance in WOLEDs based on exciplex emission, as shown in Table 3.

Figure 6b demonstrates the energy transfer mechanism of WOLEDs. The system contains three RISC-related channels: the 26DCzPPY: B4PyPPM exciplex, peaking at approximately 461 nm; the DMAC-DPS: B4PyPPM exciplex, peaking at approximately 490 nm; and the intrinsic TADF emission of DMAC-DPS, peaking at approximately 471 nm. The carrier first recombines on the high-energy excitation host of 26DCzPPY: B4PyPPM exciplex, and singlet excitons of the 26DCzPPY: B4PyPPM exciplex can transfer energy via FRET to DMAC-DPS: B4 PyPPM and PO-01 to emit light. On the other hand, triplet excitons that are not up-converted in 26DCzPPY: B4PyPPM can transfer energy to triplet excitons of DMAC-DPS. It is notable that DMAC-DPS serves as a TADF sensitizer, which promotes triplet excitons to further up-convert into singlets, and then enhances FRET from DMAC-DPS to 26DCzPPY: DMAC-DPS: B4PyPPM. On the one hand, the increased RISC process improves the RISC rate and increases the device’s efficiency. On the other hand, the efficient FRET process suppresses the trapping effect of dyes and enhances the spectral stability.

## 4. Conclusions

In summary, a blue exciplex with a triplet RISC process is developed to realize efficient hybrid white OLEDs. The additional RISC channel enhances FRET from the host to 26DCzPPY: DMAC-DPS: B4PyPPM and thus improves exciton utilization. By introducing an ultrathin PO-01 orange-emitting layer, white emission is obtained through the combination of RISC-assisted blue emission and PO-01-based orange emission. The optimized WOLED shows a maximum current efficiency of 49.1 cd/A and a maximum power efficiency of 34.8 lm/W, with a low current efficiency roll-off. Stable spectra are achieved due to the broadened recombination zone and balanced transport of the devices. This work provides a useful design principle for high-efficiency WOLEDs.

## Figures and Tables

**Table 1:** Key exciton kinetic parameters of exciplex films.

Film	Φ_PLQY_ [%]	τ_PF_ [ns]	τ_DF_ [μs]	K_ISC_ [×10^7^ s^−1^]	K_RISC_ [×10^6^ s^−1^]
26DCzPPy: B4PyPPM	23	38.4	6.86	2.58	3.62
DMAC-DPS: B4PyPPM	46	39.4	8.58	2.51	4.84
26DCzPPy: B4PyPPM: DMAC-DPS	57	46.5	9.76	2.12	5.08

Φ_PLQY_, photoluminescence quantum yield. τ_PF_, prompt fluorescence lifetime. τ_DF_, delayed fluorescence lifetime. K_ISC_, rate of intersystem crossing process. K_RISC_, rate of reverse intersystem crossing process.

**Table 2:** Performance of WOLEDs.

	V_on_ [V]	CE_max_ [cd/A]	PE_max_ [lm/W]	CIE	ΔCIE	CT (K)
W1	2.6	36.4	26.8	(0.328, 0.426)	(0.001, 0.005)	5682
W2	2.5	45.7	32.4	(0.359, 0.440)	(0.001, 0.008)	4862
W3	2.4	49.1	34.8	(0.393, 0.455)	(0.001, 0.003)	4152
W4	2.4	43.9	32.8	(0.372, 0.443)	(0.005, 0.002)	4560

V_on_ [V]: turn-on voltage; CE_max_ [cd/A]: maximum CE; PE_max_ [lm/W]: maximum PE; CIE: CIE coordinates at 7 V; ΔCIE: CIE coordinates variation from 5 V to 8 V; CT: color temperature at 7 V.

**Table 3:** Summary of EL performance of WOLEDs based on exciplex emission.

	V_on_ (V)	CE_max_ [cd/A]	PE_max_ [lm/W]	CIE
This work	2.4	49.1	34.8	(0.393, 0.455)
[20]	2.9	55.3	46.8	(0.47, 0.49)
[21]	2.6	34.4	36.1	(0.393, 0.455)
[24]	3.6	19.3	16.9	(0.33, 0.44)
[25]	3.3	13.8	11.6	(0.351, 0.402)

V_on_ [V]: turn-on voltage; CE_max_ [cd/A]: maximum CE; PE_max_ [lm/W]: maximum PE; CIE: CIE coordinates.

## Data Availability

The data that supports the findings of this study are available on request from the corresponding authors.
